# PgtE Enzyme of *Salmonella enterica* Shares the Similar Biological Roles to Plasminogen Activator (Pla) in Interacting With DEC-205 (CD205), and Enhancing Host Dissemination and Infectivity by *Yersinia pestis*

**DOI:** 10.3389/fimmu.2022.791799

**Published:** 2022-03-24

**Authors:** Qiao Li, Chenglin Ye, Fei Zhao, Wenjin Li, Sizhe Zhu, Yin Lv, Chae Gyu Park, Yingmiao Zhang, Ling-Yu Jiang, Kun Yang, Yingxia He, Huahua Cai, Song Zhang, Hong-Hui Ding, Olivia Adhiambo Njiri, John Mambwe Tembo, Ayman Ahmad Alkraiem, An-Yi Li, Zi-Yong Sun, Wei Li, Mei-Ying Yan, Biao Kan, Xixiang Huo, John D. Klena, Mikael Skurnik, Andrey P. Anisimov, Xiaofang Gao, Yanping Han, Rui-Fu Yang, Xiding Xiamu, Yuanzhi Wang, Hongxiang Chen, Bao Chai, Yicheng Sun, Jingping Yuan, Tie Chen

**Affiliations:** ^1^Tongji Hospital, Tongji Medical College, Huazhong University of Sciences and Technology, Wuhan, China; ^2^Department of Pathology, Renmin Hospital of Wuhan University, Wuhan, China; ^3^Therapeutic Antibody Research Center, Genuv Inc., Seoul, South Korea; ^4^Immune and Vascular Cell Network Research Center, National Creative Initiatives, Department of Life Sciences, Ewha Womans University, Seoul, South Korea; ^5^Union Hospital, Tongji Medical College, Huazhong University of Sciences and Technology, Wuhan, China; ^6^Tongji Hospital, Tongji Medical College, Huazhong University, Wuhan, China; ^7^Department of Biology, College of Science, Taibah University, Medina, Saudi Arabia; ^8^National Institute for Communicable Diseases Control and Prevention, Chinese Center for Disease Control and Prevention, Beijing, China; ^9^Center for Infectious Diseases, Hubei Provincial Centers for Disease Control and Prevention (CDC), Wuhan, China; ^10^Viral Special Pathogens Branch, Centers for Disease Control and Prevention, Atlanta, GA, United States; ^11^Department of Bacteriology and Immunology, University of Helsinki, Helsinki, Finland; ^12^Laboratory for Plague Microbiology, State Research Center for Applied Microbiology and Biotechnology, Obolensk, Russia; ^13^State Key Laboratory of Pathogen and Biosecurity, Beijing Institute of Microbiology and Epidemiology, Beijing, China; ^14^Division of Disease Control and Prevention for Endemic Diseases , Wenquan Center for Disease Control and Prevention, Wenquan, China; ^15^Department of Pathogen Biology and Immunology, Shihezi University School of Medicine, Shihezi, China; ^16^Department of Dermatology, Huazhong University of Science and Technology Union Shenzhen Hospital, Shenzhen, China; ^17^Department of Dermatology, The 6th Affiliated Hospital of Shenzhen University Health Science Center, Shenzhen, China; ^18^Ministry of Health (MOH) Key Laboratory of Systems Biology of Pathogens, Institute of Pathogen Biology, Chinese Academy of Medical Sciences and Peking Union Medical College, Beijing, China

**Keywords:** *Yersinia pestis*, *Salmonella enterica*, DEC-205 (CD205), PgtE, dissemination, evolution

## Abstract

*Yersinia pestis*, the cause of plague, is a newly evolved Gram-negative bacterium. Through the acquisition of the plasminogen activator (Pla), *Y. pestis* gained the means to rapidly disseminate throughout its mammalian hosts. It was suggested that *Y. pestis* utilizes Pla to interact with the DEC-205 (CD205) receptor on antigen-presenting cells (APCs) to initiate host dissemination and infection. However, the evolutionary origin of Pla has not been fully elucidated. The PgtE enzyme of *Salmonella enterica*, involved in host dissemination, shows sequence similarity with the *Y. pestis* Pla. In this study, we demonstrated that both *Escherichia coli* K-12 and *Y. pestis* bacteria expressing the PgtE-protein were able to interact with primary alveolar macrophages and DEC-205-transfected CHO cells. The interaction between PgtE-expressing bacteria and DEC-205-expressing transfectants could be inhibited by the application of an anti-DEC-205 antibody. Moreover, PgtE-expressing *Y. pestis* partially re-gained the ability to promote host dissemination and infection. In conclusion, the DEC-205-PgtE interaction plays a role in promoting the dissemination and infection of *Y. pestis*, suggesting that Pla and the PgtE of *S. enterica* might share a common evolutionary origin.

## Introduction

*Yersinia pestis*, a Gram-negative bacterium, is the causative agent of bubonic, septicemic, and pneumonic plague (1). *Y. pestis* has been responsible for all three historical plague pandemics, including the Justinian, the Black Death, and the third pandemic (2–4), as well as one or more prehistoric plague pandemic (5, 6). The study by Rascovan et al. revealed a prehistoric plague pandemic between 6,000–5,000 BP that occurred at the same time with the decline of Neolithic populations in Europe and suggested that this pandemic caused by multiple lineages of *Y. pestis* expanded across Eurasia might result in the decline (6). As also summarized by the author, “Our results are consistent with the existence of a prehistoric plague pandemic that likely contributed to the decay of Neolithic populations in Europe” (6). This bacterial pathogen was also used as a biological weapon during the Second World War (7).

*Y. pestis* evolved from *Yersina pseudotuberculosis* within the last 2,600 to 28,000 years (4, 8–11), but each of them causes very different diseases in animals. *Y. pseudotuberculosis* typically transmitted through the fecal-oral route and primarily causes mesenteric lymphadenitis and self-limited diarrhea in the host (12). In contrast, *Y. pestis* causes in the host a highly fatal disease, known as the plague, in the host (1). Many comparative studies between *Y. pestis* and *Y. pseudotuberculosis* have been carried out to determine what are the virulence factors *Y. pestis* has acquired during evolution that have converted the mild pathogen *Y. pseudotuberculosis* to a highly virulent and deadly pathogen. Notably, the plasmid pPCP1 was one of them.

The plasmid pPCP1 was acquired by an ancestral strain after Pestoides F *Y. pestis* (do not carry pPCP1) in the evolution tree, during the divergence from *Y. pseudotuberculosis* into modern *Y. pestis* (13–16). One key factor that promotes *Y. pestis* pathogenesis is plasminogen activator (Pla), which is encoded by the pPCP1 plasmid (17). Zimbler et al. even speculated that the ancestral strain, Pestoides F, was unable to cause primary pneumonic plague prior to the acquisition of Pla (16). Pla is required for the full virulence of *Y. pestis* in both bubonic and pneumonic plague but is not essential for the pathogenesis of septicemic plague (18–24). These results have indicated that Pla may facilitate the dissemination of *Y. pestis* within hosts.

Studies have shown that Pla promotes the fibrinolysis, allowing the bacteria to disrupt tissue barriers at the subdermal injection sites either after a flea bite or experimental subdermal inoculation. This process facilitates the bacterial dissemination into the lymphatic tissue, liver, and spleen of the host (19, 21, 24). Because Pla belongs to a family of enteric bacterial outer membrane proteases, the bacterial species that initially harbored the Pla-encoding gene is thought to most likely be an enteric bacterium.

Sodeinde and Goguen reported sequence homology among the Pla, OmpT, expressed by *Y. pestis* and *Escherichia coli* and the PgtE enzyme expressed by *Salmonella enterica*, which causes mouse typhoid (25). The DNA sequence identity between the *pla* and *pgtE* genes within the coding regions is 69% (25).

PgtE is known to be involved in the host dissemination of *S. enterica* (26). PgtE can degrade gelatine and activate matrix metalloproteinase 9 (26) to enhance bacterial motility. The deletion of the *pgtE* gene from *S. enterica* resulted in a ten-fold reduction in bacterial dissemination within hosts to the liver and the spleen following intraperitoneal infection in BALB/c (26). Although other scientists began to observe that the dissemination of *S. enterica* within the host involves a constant phagocytosis process by antigen presenting cells (APCs) such as macrophages and dendritic cells (27–31), the molecular mechanism through which PgtE promotes bacterial dissemination in hosts has been thought to be associated with its ability to disrupt adjacent tissues too.

However, the results from a 2008 study appeared to challenge this accepted mechanism by reporting that the Pla of *Y. pestis* interacted with a C-type lectin, DEC-205 (CD205), which is typically expressed on antigen-presenting cells (APCs). By Pla-mediated binding to DEC-205, *Y. pestis* might be able to hijack alveolar macrophages or lung dendritic cells, acting as Trojan horses to facilitate dissemination from the lungs to the spleen (32). DEC-205 was originally identified as a strong antigen-presenting receptor by Nussenzweig and Steinman’s group (33–35). By conjugating with other antigenic proteins, such as the proteins expressed by pancreatic cancers, the hybridized DEC-205 displayed a strong adjuvant effect on the host immune response to pancreatic cancers (36, 37).

Although sequence comparisons have suggested the possibility that Pla in *Y. pestis* might have derived from PgtE in *Salmonella enterica* (25), no direct evidence has been reported to support any functional links between PgtE and Pla. Based on the findings published on Journal of Biological Chemistry in 2008 (32), we were using a similar approach to investigate whether the C-type lectin receptor CD205 would also bind to PgtE from *S. enterica* to facilitate host dissemination and bacterial infection in *Y. pestis*. The results from this study might help us a further understanding of how *Y. pseudotuberculosis* that causes mild mesenteric lymphadenitis and self-limited diarrhea has evolved to such a deadly and distinctive pathogen, *Y. pestis*.

## Materials and Methods

### Ethics Statement

All animal procedures and human experiments were conducted in strict accordance with the Institutional Animal Care and Use Committees (IACUCs) and Institutional Review Board (IRB) of Tongji Hospital, Tongji Medical College, China. The handling of mice and all experimental procedures were specifically approved for this study by the Medical Ethics Committee of Tongji Hospital and were performed in accordance with institutional guidelines (IRB ID: TJ-A20141220 for animal experiments). All procedures on mice were performed under sodium pentobarbital anesthesia; all efforts were made to minimize animal suffering.

### Mice

C57BL/6J mice, aged 6–8 weeks, were purchased from Wuhan University Animal Center, China. Mice were housed in the animal facilities at the Tongji Hospital, in direct accordance with the guidelines drafted by the Animal Care Committees of Tongji Hospital.

### Bacterial Strains

Bacterial strains used in this study were listed in **Table 1**. *Y. pseudotuberculosis* Y1 is a strain that lacks the virulence plasmid (pYV) and was used as a positive control in the cell invasion assay in previous publications, because this strain appears invading almost all mammalian cells lines (32, 40–46).

**Table 1:** Bacteria strains and cell lines used in the study.

Strains	Genotypes	References	
***Y. Pseudotuberculosis***			
***Y. pestis***			
*Y.p*1418-Δail	Originated from KIM5 (KIM D27) with *pgm* (pigmentation) and *ail* gene deleted	(6)	
*Y.p*1419	a derivative of *Y.p*1418, Originated from KIM5 (KIM D27) with *pgm*, *pla* and *ail* gene deleted	this study	
*Y.p*1419 pPCP1*^+^*	a derivative of *Y.p*1418,*Y.p*1418 transformed with pPCP1 plasmid, with ampicillin antibiotic resistance	this study	
*Y.p*1419 *pla^+^*	a derivative of *Y.p*1418,*Y.p*1418 transformed with plasmid carrying pla expressing gene of *Y.pestis*, with ampicillin antibiotic resistance	this study	
*Y.p*1419 *pgtE^+^*	a derivative of *Y.p*1418, *Y.p*1418 transformed with pgtE expressing gene of *Salmonella*, with ampicillin antibiotic resistance	this study	
*Y.p*91001	a human avirulent *Y.pestis* strain F1+, LcrV+, Pst+ and Pgm+ isolated from Microtus-related plague focus in China	(38, 39)	
*Y.p*91001*pla^-^*	91001 *Y. pestis* strain with *pla* gene deleted	From Yicheng Sun	
*Y.p*91001*pla^-^+pla^+^*	91001 *Y. pestis* strain with *pla* gene deleted and restored with the Pla expression, with ampicillin antibiotic resistance	thiss study	
*Y.p*91001*pla^-^+pgtE^+^*	91001 *Y. pestis* strain with *pla* gene deleted and restored with the PgtE expression, with ampicillin antibiotic resistance		this study
*Y.p* 91001pPCP1^-^	91001 *Y. pestis* strain with pPCP1 plasmid cured	this study	
*Y.p* 91001pPCP1^-^*pla^+^*	91001-pPCP1^-^ restored with the plasmids pMRK1 encoding *Y. pestis* Pla, with ampicillin antibiotic resistance	this study	
*Y.p* 91001pPCP1^-^*pgtE^+^*	91001-pPCP1^-^ restored with the plasmids pMRK3 encoding *Salmonella* PgtE, with ampicillin antibiotic resistance	this study	
***E. coli* K-12**			
*E. coli*	Wide type *E. coli*		
*E. coli* *pla+*	*E. coli* XL1 transformed with the plasmids pMRK1 encoding *Y. pestis* Pla, with ampicillin antibiotic resistance	(27)	
*E. coli pgtE^+^*	*E. coli* XL1 transformed with the plasmids pMRK3 encoding *Salmonella* PgtE, with ampicillin antibiotic resistance	(27)	
**Cell lines**	**Characteristics**		
CHO-NEO cells	Control cell line, which expresses the neomycin resistance gene only	(40, 41)	
CHO-m-DEC205 cells	Generated by transfecting CHO cells with CD205 cDNAs	(6)	
Mouse alveolar macrophages	Primary macrophages from mouse alveolar		

*Y*. *pestis* 1418 originates from KIM5 (KIM D27), a strain from which the *pgm* (pigmentation) locus was deleted (45) and it therefore is bio-safety level II strain. In this study, the virulence plasmid (pCD1) and the *ail* gene were also deleted (32, 41). There are two purposes for construction of this non-virulence *Y. pestis.* First, one of the important functions of Ail is to mediate *Y. pestis* attachment to and invasion into the host cells (47, 48). The second reason was for the biosafety issues set by our regulators. Strain *Y.p*1419 originates from *Y. pestis* 1418 but features the additional deletion of pPCP1 plasmid. *Y. pestis* strains *Y.p*1419 *pla*^+^ and *Y.p*1419 *pgtE*^+^ are derivatives of *Y. pestis* 1419 that carries plasmids pMRK1 and pMRK3 that express the *Y. pestis* Pla and *Salmonella* PgtE.

*Y. pestis Y.p*91001 was isolated from Microtus-related plague focus in China, and is avirulent to humans but can cause plague in rodents belonging to the genus *Microtus* and laboratory mice (45, 49–51). *Y. p* 9100pPCP1^-^ is *Y. pestis* 91001, from which the plasmid pPCP1 has been cured. *Y. p*91001pPCP1^-^pla^+^ is *Y.p*91001-pPCP1^-^ restored with the plasmids pMRK1 encoding *Y. pestis* Pla. *Y. p*91001pPCP1^-^*pgtE*^+^is *Y.p*91001-pPCP1^-^ restored with the plasmids pMRK3 encoding *Salmonella* PgtE.

*E. coli pla^+^* carries the plasmid pMRK1 that expresses the Pla of *Y. pestis*. *E. coli pgtE^+^* carries the plasmid pMRK3 that expresses the *Salmonella* PgtE.

The *Y. pestis* is cultured at 26°C in Luria-Bertani (LB) for 48 hours with shaking to log phase. The *E. coli* is cultured at 37°C in Luria-Bertani (LB) overnight with shaking to log phase.

The plasmid pMRK1 was constructed by cloning complete opening reading frame of pla from plasmid pC4006 into pSE380 plasmid (52). The source of Pla sequences for transgene expression is *Y. pestis* KIM5 pgml (spontaneous non pigmented mutant of *Y. pestis* KIM) (25).

The source of PgtE sequences for transgene expression was from the genomic DNA of *S. enterica* SH401 (database accession number AF239770) (53). The plasmid pMRK3 was constructed by cloning the complete reading frame of pgtE from *S. enterica* SH401 into pSE380 plasmid (53, 54).

### Generation of the *pla-*Knockout of *Y. pestis* 91001

For generating the *pla* knockout strain, CRISPR-Cas12a system were used to delete the *pla* gene in the plasmid pPCP1 of *Y. pestis*, following the protocol that worked on other *Y. pestis* strains (55). Briefly, a protospacer adjacent motif (PAM) TTC and a short DNA sequence adjacent to the protospacer site were selected from the coding sequence of the *pla* gene. Two complementary oligonucleotides (crRNA-pla top and crRNA-pla bottom) containing the protospacer sequence were synthesized, annealed to yield a protospacer cassette with BsaI overhangs at the 5’and 3’ends. Then the protocpacer was cloned into the crRNA expression plasmid pAC-crRNA-Cm to generate the recombinant plasmid pcrRNA-pla-Cm. An 80 nt ssDNA oligonucleotide (pla oligo for lagging) with identity to flanking regions sequence on both sides of the *pla* gene was synthesized. The ssDNA oligonucleotides and the recombinant plasmid pcrRNA-inv-Cm were co-transformed into *Y. pestis* cells harboring the plasmid pKD46-cpf1 (Cas12a). The transformants were plated on LB agar supplemented with 100 μg/mL ampicillin and 30 µg/mL chloramphenicol and incubated at 26°C Single colonies were picked up to inoculate LB medium supplemented with appropriate antibiotics at 26°C. PCR was used with appropriate primers to carry out preliminary screen, and sequencing to verify the *pla* deletion clones. The primers used in this study were listed in **Table 2**. Finally, the plasmids pcrRNA-pla-Cm and pKD46-cpf1 were cured from the strain by incubating on LB agar plate with supplemented with 7% of sucrose, and by incubating in LB medium at 42°C with shaking overnight, respectively. The plasmids used in this study were listed in **Table 3**.

### Biological Reagents

Human Glu-Plasminogen was purchased from Hematologic Technologies (Essex Junction, VT, USA), and the chromogenic plasmin substrate S-2251 was purchased from Chromogenix (Milano, Italy). Anti-human CD205 antibodies were purchased from Pharmingen (San Diego, CA, USA).

### Cell Lines

CHO-m-DEC205 was generated by transfecting CHO cells with the corresponding human C-type lectin cDNA. Transfected cells were selected by G418 (1.5 mg/ml) and screened for the stable surface expression of CD205. CHO-NEO, which expresses the neomycin resistance gene without other exogenous genes, was used as the control cell line (32). Cells were cultured in RPMI supplemented with 10% fetal calf serum (FCS), streptomycin (100 μg/ml), and penicillin (100 units/ml) and incubated at 37°C with 5% CO_2_.

### Isolation of Mouse Alveolar Macrophages

C57BL/6J mice, aged 6–8 weeks, were anesthetized (32). Alveolar macrophages were obtained using the following procedures. After the mice were euthanized, the bronchial tract was opened, the upper bronchia tract was ligated with surgical suture and 1 ml of RPMI medium was injected into the lungs through a syringe. The mouse chest was gently massaged for 3 mins, and then the lavage fluid was collected. The cell number in the lavage fluid was adjusted to 1×10^5^ cell/ml. A glass-slide was plated in the 24-wells plate before seeding the cells. The macrophages were seed into 24-wells plates at the concentration of 1×10^5^ cell/well and placed in RPMI medium with 2% FBS in a CO_2_ incubator for 2 h. The cell layers were washed three times to remove non-adherent cells.

### Cell Invasion Assay

The cell invasion assays were performed as described previously (43, 44). CHO and CHO-m-DEC205 cells were suspended in RPMI 1640 medium supplemented with 2% FCS. Cells were plated in 24-wells plates at a density of 1 × 10^5^ cells/well in 0.5 ml medium. Then, 50 µl of bacterial suspensions were added at a concentration of 1 × 10^7^ colony-forming units (CFU)/ml,5×10^5^CFU in total. *Y. pestis* was centrifuged at 500 rpm for 5min to initiate the *in vitro* infection. The cells were incubated with the bacteria at 37°C for 2.5 h with 5% CO2. The cells were washed with phosphate-buffered saline (PBS) three times. To kill any extracellular bacteria, 2 ml RPMI-1640 containing 2% fetal bovine serum (FBS) containing gentamycin, at 100 μg/ml, was added to each well and incubated for 1 hour. The cells in the 24-well plate were washed twice with PBS, and then the cells were lysed with 1 ml 1% Triton X-100. The cell lysates were diluted and plated on LB agar plates. The bacterial colonies recovered from the lysed cells were counted after 2 days to define the level of internalized bacteria in the host cells. All experiments were performed in triplicate, and the data were expressed as the mean ± standard error.

### Plasminogen Activation Assay

Plasminogen activation was measured as described in previously published studies (46, 52, 53). Briefly, 8×10^7^ of bacteria were suspended in PBS and combined with 4 μg human Glu-Plasminogen and 0.45 mM S-2251 in 96-well plates, at a final volume of 200 µl, followed by incubation at 37°C. The absorption values at 405 nm were measured at 30-min intervals. The results are presented as the difference between each measurement value and the starting value.

### Animal Challenge for Bacterial Dissemination

*Y. pestis* were cultured at 27°C and suspended in PBS at an OD_600_ = 1, resulting in the retrieval of 3 ml bacterial suspension (32). C57BL/6J mice, 6–8 weeks of age, 5 mice in each group, were inoculated with 20 µl bacterial suspension, 2*10^7^ CFU in total, *via* the intranasal route using the following bacteria: *Y.p*91001, *Y.p*91001pPCP1^-^, *Y.p*91001pPCP1^-^*pla*^+^ and *Y.p*91001 pPCP1^-^*pgtE*^+^, and another panel of bacteria *Y.p*91001, *Y.p*91001*pla*-, *Y.p*91001*pla^-^* +*pla^+^*, *Y.p*91001*pla^-^* +*pgtE^+^*. It is should be stated that all the strains of *Y. pestis* used here were the virulence plasmid (pYV) -cured derivatives of *Y. pestis*. After 3 days of infection, the liver and spleen were collected and homogenized in 1% Triton X-100 to release the bacteria. The tissue lysate was plated onto LB agar plates supplemented with 50μg/ml ampicillin as shown in our previous publications (41, 45, 46). The dissemination rate was calculated by determining the CFU recovered from the lysed tissue samples.

### Animal Challenge for Survival

*Y. pestis* 91001 were cultured at 27°C, collected by centrifugation, and suspended in PBS at OD_600_ = 1, resulting in the collection of 3 ml bacterial suspension following centrifugation (45). C57BL/6J mice, aged 6-8 weeks, were infected with 20 µl of the various *Yersinia* suspensions, 2*10^7^ CFU in total, *via* the intranasal route. Ten mice in each group were infected by two sets of bacteria, including *Y.p*91001, *Y.p*91001 pPCP1^-^, *Y.p*91001 pPCP1^-^*pla*^+^ and *Y.p*91001 pPCP1^-^*pgtE*^+^ strains. The death of the mice was recorded every 12 h for 12 days.

### Histopathological Studies

Samples from either the dissemination or survival assays were fixed in 4% neutral buffered formalin. Tissue embedding, sectioning, and staining with hematoxylin and eosin (H&E) were performed by the Servicebio biological laboratory. Stained sections were analyzed under a light microscope.

### Statistical Analyses

All statistical analyses were completed using Prism software, version 6 (Graph Pad, San Diego, CA, USA). Significance was assessed using ANOVA test. Survival group comparisons were performed *via* the log-rank test using Kaplan–Meier analysis. P<0.05 was considered to be the threshold for significance.

## Results

### PgtE Expressed in *Y. pestis* Can Activate Plasminogen Into Plasmin

**Figure 1:**
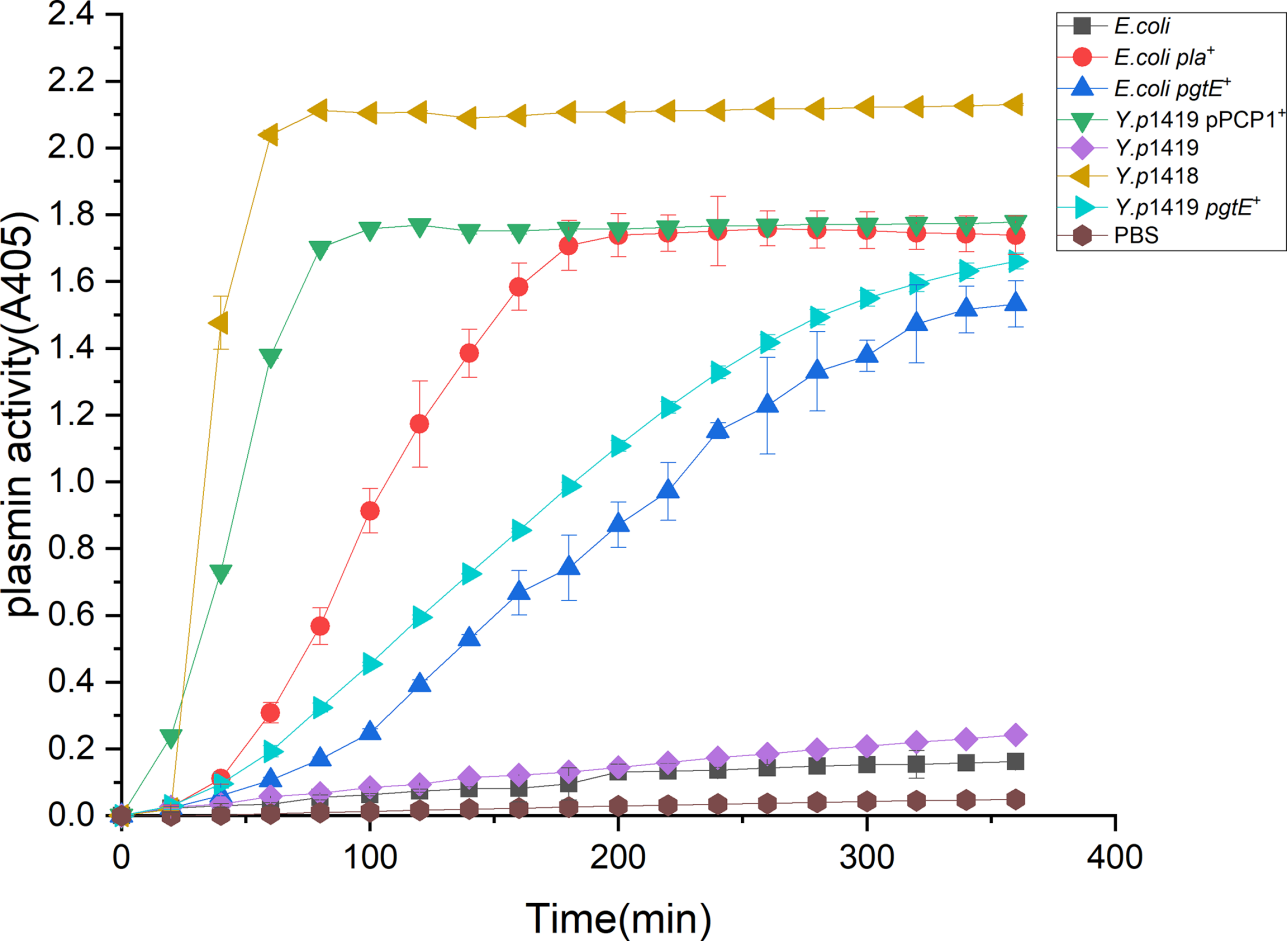
PgtE in recombinant *Y. pestis* activates plasminogen to plasmin. The plasminogen activation activities of *Y.p*1419 pPCP1^+^*, Y.p*1419, *Y.p*1419 *pgtE^+^* and *E.coli, E.coli pla^+^* were compared. *Y.p*1418 was used as a positive control. PBS was used as negative control. The data presented were pooled from three independent experiments.

One proposed mechanism for the dissemination of *Y. pestis* depends on the plasminogen activator Pla, which can degrade fibrous connections in the tissue matrix. We examined whether *Y. pestis* strains that express PgtE of *S. enterica* could activate plasminogen to plasmin similar to the action of Pla. Plasmids expressing Pla and PgtE were transformed into the *Y. pestis* strain 1419 that does not contain the pPCP1 plasmid. We compared the plasminogen activation activity among *Y. p*1419 pPCP1^+^*, Y.p*1419*, Y.p*1419 *pgtE^+^* and *E.coli, E.coli pla^+.^*, *Y.p*1418, which expresses Pla, were used as positive controls (53). *Y. p*1419 was used as the negative control. As shown in **Figure 1**, *Y.p* 1418 showed the highest plasminogen activation activity among the examined strains. *Y.p*1419 pPCP1^+^ showed higher plasminogen activation activity than *Y.p*1419 *pgtE^+^* (53). Consistent with previous studies, this result also indicated that Pla induced higher plasminogen activation activity than PgtE did.

These data demonstrated that *Y. pestis* Pla and *S. enterica* PgtE shared similar functions with regarding to the activation of plasminogen into plasmin, suggesting that *Y. pestis* Pla might have evolved from *S. enterica* PgtE.

### PgtE-Expressing *E. coli* and *Y. pestis* Enhance Phagocytosis by Primary Alveolar Macrophages and Invade CHO-DEC-205

Our previous study demonstrated that Pla in *Y. pestis* could promote the invasion of alveolar macrophages, mediated by the interaction with the C-type lectin receptor CD205 (32). Therefore, we tested the invasion of alveolar macrophages by two panels of bacteria, including *E. coli, E. coli pla^+^*, *E. coli pgtE^+^* and *Y.p*1418*, Y.p*1419*, Y.p*1419 *pla^+^, Y.p*1419 *pgtE^+^*.

**Figure 2:**
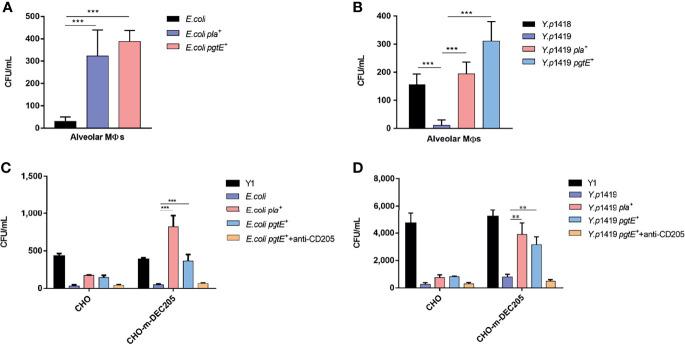
PgtE-expressing *E. coli* and *Y. pestis* invade alveolar macrophages and invade CHO-m-DEC-205. **(A)** PgtE-expressing *E. coli* were examined for their ability to enter alveolar macrophages. The bacteria used were *E. coli, E. coli pla^+^* and *E. coli pgtE+*. **(B)** PgtE-expressing *Y. pestis* were examined for their ability to enter alveolar macrophages. The bacteria used were *Y.p*1418*, Y.p*1419*, Y.p*1419 *pla^+^, Y.p*1419 *pgtE^+^*.The number of phagocytized bacteria was determined by evaluating the CFUs on the plates after two days. The data presented were collective from three independent experiments. *P < 0.05, **P < 0.01, ***P < 0.001. **(C)** PgtE-expressin*g E. coli* invade the CHO cell line expressing CD205. Epithelial CHO cells expressing or not expressing CD205 (CHO and CD205, respectively) were infected with PgtE- and Pla-expressing *E. coli*. *Y. pseudotuberculosis* (Y1); *E. coli, E. coli pla^+^*, and *E. coli pgtE^+^* were examined for their abilities to invade CHO/CHO-m-DEC-205 cells during a gentamicin protection assay, in presence or absence of anti-DEC-205 (5 μg/ml). The numbers of phagocytosed bacteria were determined by counting the bacterial CFUs on the plates the next day. The data presented were pooled from three independent experiments. *P < 0.05, **P < 0.01, ***P < 0.001. **(D)** PgtE-expressin*g Y. pestis* invades the CHO cell line expressing CD205. Epithelial CHO cells expressing or not expressing CD205 (CHO and CD205, respectively) were infected with PgtE- and Pla-expressing *Y. pestis*. *Y. pseudotuberculosis* (Y1), *Y.p*1419*, Y.p*1419 *pla^+^* and *Y.p*1419 *pgtE^+^* were examined for their abilities to invade CHO/CHO-m-DEC-205 cells during a gentamicin protection assay, in presence or absence of anti-DEC-205 (5 μg/ml). The numbers of phagocytosed bacteria were determined by counting the bacterial CFUs on the plates after two days. The data presented were pooled from three independent experiments. *P < 0.05, **P < 0.01, ***P < 0.001.

As shown in **Figure 2**, we found that the both Pla- and PgtE-expressing *Y. pestis* and *E. coli* were able to enhance the invasion of bacteria into mouse alveolar macrophages. Previous studies have indicated that mouse alveolar macrophages express a C-type lectin DEC-205, which could be hijacked by *Y. pestis via* PLA, leading to dissemination within the host (32, 58). It was therefore investigated whether CD205 plays a role in the phagocytosis of recombinant *E. coli* and *Y. pestis* pgtE *via* alveolar macrophages.

(32), We further explored whether this interaction was mediated by the binding between PgtE and CD205 on mouse alveolar macrophages. The results shown in **Figures 2C, D** demonstrated that the CHO-mDEC-205 cells were able to phagocytize PgtE-expressing *E. coli* and *Y. pestis. E. coli pla^+^* was used as a control for its ability to interact with DEC-205 in **Figure 2C** (32). This CD205-PgtE interaction was inhibited by anti-CD205 antibody (**Figures 2C, D****)**. *Y. pseudotuberculosis* was used as another control for its invasion of almost all epithelial cells (32, 38, 45, 46).

Based on the above evidence, we conclude that CD205 serves as a receptor for PgtE and contributes to the phagocytosis of PgtE-expressing *E. coli* and *Y. pestis*, indicating that PgtE and Pla share a similar ability to binding to the C-type lectin receptor CD205. The inhibition of the interaction by adding the anti-CD205 antibody supports that the binding is specific, and that CD205 functions as a receptor in the interaction between CD205 and pgtE-expressing *E. coli* and *Y. pestis*.”

### PgtE Expressed in *Y. pestis* Confers the Ability to Promote Host Dissemination

**Figure 3:**
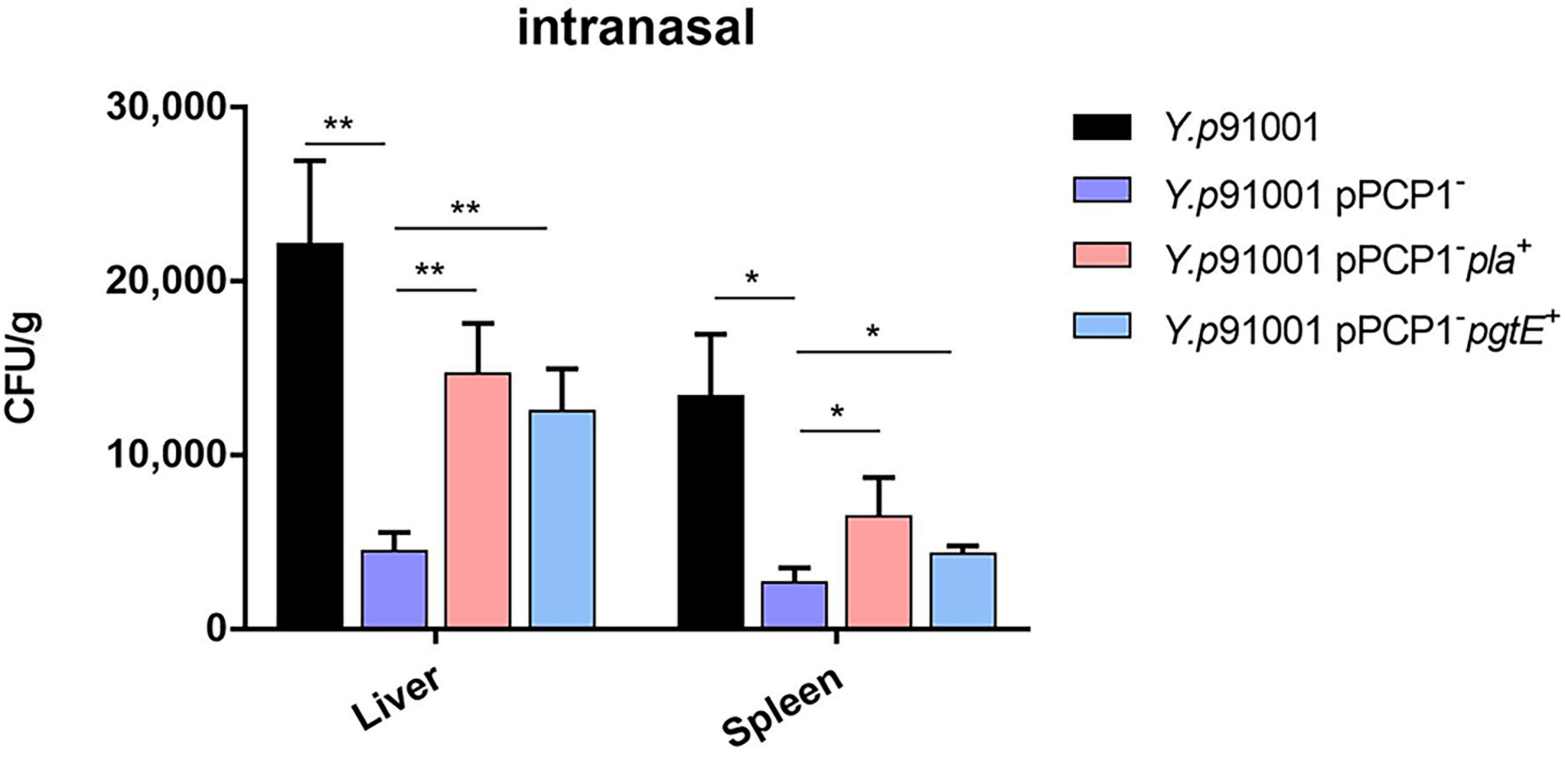
PgtE expressed in *Y. pestis* enhances the ability to promote host dissemination. *Y.p*91001, *Y.p*91001pPCP1^-^, *Y.p*91001 pPCP1^-^*pla*^+^ and *Y.p*91001 pPCP1^-^*pgtE*^+^ were used to challenge mice *via* the intranasal route. After 72 hours of infection, the liver and spleen were collected and homogenized. The bacterial loads were quantified by counting the bacteria colonies on the plates after two days. (The data shown were obtained from three independent experiments. *P < 0.05, **P < 0.01.

We showed previously that Pla in *Y. pestis* could bind to CD205 to facilitate the dissemination of *Y. pestis* (32). Therefore, we hypothesized that the dissemination of *Y. pestis* to the spleen and liver would also be facilitated by the PgtE-CD205 interaction. To mimic evolution, we chose a wild-type and *Y. pestis* strain 91001 that contains the pigmentation locus, *pgm*, and the plasmids pCD1 and pPCP1 (**Figure 3**) (49).

C57BL/6J mice were inoculated *via* the intranasal route with *Y. pestis Y.p*91001, its isogenic strain that lacks Pla (*Y.p*91001pPCP1^-^), the pPCP1^-^strain complemented with the coding sequence for Pla (*Y.p*91001pPCP1^-^*pla*^+^), and the pPCP1^-^strain complemented with the coding sequence for PgtE (*Y.p* 91001pPCP1^-^*pgtE*^+^).

The dissemination rates of bacteria into the different organs were calculated by counting the CFUs on the plates. **Figure 3** shows that the CFU numbers of 91001pPCP1^−^ were lower than the other strains that were isolated from the liver and spleen. Furthermore, both *Y.p*91001pPCP1^-^*pla*^+^ and *Y.p*91001pPCP1^-^*pgtE*^+^ were able to disseminate to the liver and spleen. In short, the results suggested that PgtE conferred the ability to promote host dissemination of *Y. pestis*.

### The Expression of PgtE in *Y. pestis* Enhances the Fatality Rate in Mice

CD205 appeared to participate in the *in vitro* interaction of PgtE-expressing *E. coli* or *Y. pestis* with APCs; however, whether this interaction also occurs *in vivo* remained to be elucidated. C57BL/6J mice were inoculated *via* the intranasal route with *Y. pestis Y.p*91001, *Y.p*91001pPCP1^-^, *Y.p*91001 pPCP1^-^*pla*^+^ and *Y.p* 91001 pPCP1^-^*pgtE*^+^. The survival of the mice infected with these various *Y. pestis* strains was monitored. In addition, we knocked out *pla* in the plasmid of pPCP1 (*Y.p* 91001pPCP1^−^) and introduced pMRK1 (the plasmid vector pSE380 carrying *pla*) and pMRK3 (the plasmid vector pSE380 carrying *pgtE*).

**Figure 4:**
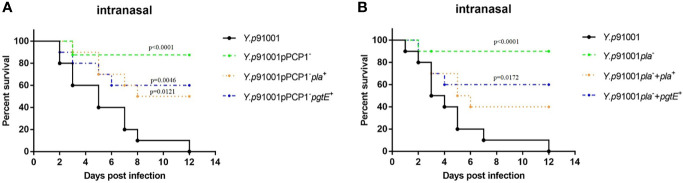
Mice infected intranasally with PgtE-expressin*g Y. pestis* are more susceptible to death compared with pPCP1 plasmid cured and pla-deleted *Y. pestis*. **(A)** *Y.p*91001, *Y.p*91001pPCP1^-^, *Y.p*91001 pPCP1^-^*pla*^+^ and *Y.p*91001 pPCP1^-^*pgtE*^+^ were used to challenge mice *via* the intranasal route. The mice were monitored for 12 days, and the log-rank test was performed. **(B)** *Y.p*91001, *Y.p*91001*pla*-, *Y.p*91001*pla^-^* +*pla^+^*, *Y.p*91001*pla^-^* +*pgtE^+^* were used to challenge mice *via* the intranasal route. The data shown were obtained from three independent experiments.

As shown in **Figure 4A**, we found that mice infected with wild-type *Y. pestis* 91001 succumbed to the infection. Curing of the pPCP1 from *Y. pestis* 91001 reduced the virulence, which is consistent with the finding from Lathem et al., who reported that the inhibition of Pla expression prolonged the survival of animals (20). We next restored the expression of Pla or PgtE using specific plasmids, which rescued the virulence of *Y. pestis.* However, compared with the Pla^+^ bacteria, their PgtE^+^ isogenic variants showed significantly lower virulence.

In **Figure 4B**, *Y.p*91001, *Y.p*91001*pla*-, *Y.p*91001*pla^-^* +*pla^+^*, *Y.p*91001*pla^-^* +*pgtE^+^* were administered to mice by intranasal challenge. We found that the absence of *pla* attenuated in a mouse model of pneumonic plague infected by *Y. pestis* 91001. Restoring Pla expression in *Y.p*91001*pla*- can almost restore the virulence to Pla(-) mutant. Overexpression of PgtE from plasmid alone can also increase the mortality rate of mice. We have shown that PgtE played a critical pathogenic role in mice succumbed to respiratory infection.

We concluded that PgtE-promoted bacterial dissemination and virulence in host may be due to in part the ability of PgtE to interact with CD205.

### PgtE-Expressing *Y. pestis* Increases Lung Tissue Inflammation

**Figure 5:**
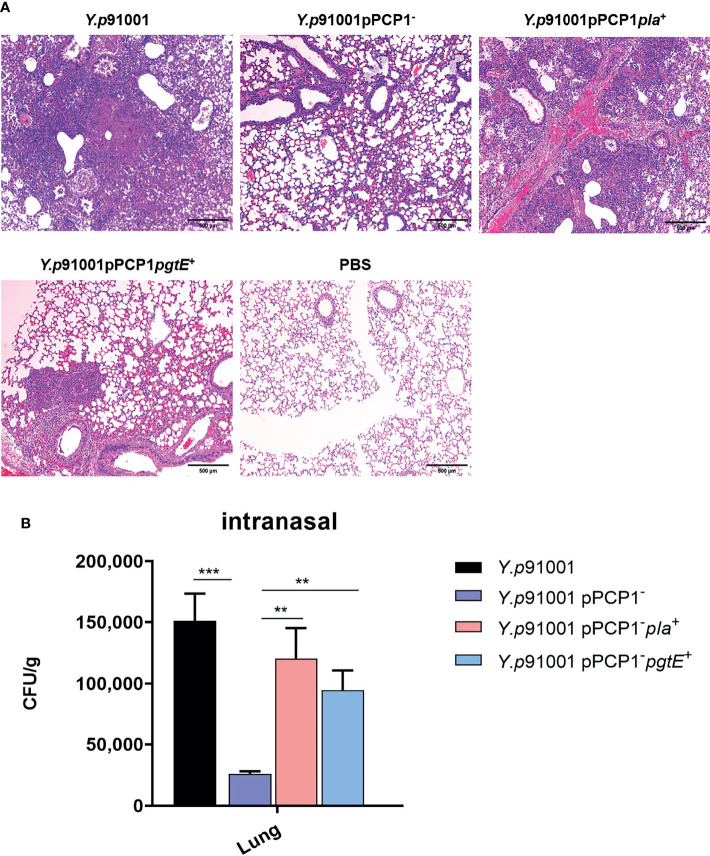
The expression of PgtE in *Y. pestis* was able to enhance the inflammatory lesions in the lungs from C57BL/6 mice. **(A)***Y.p*91001, *Y.p*91001pPCP1^-^, *Y.p*91001 pPCP1^-^*pla*^+^ and *Y.p*91001 pPCP1^-^*pgtE*^+^ were used to challenge mice *via* the intranasal route. Lung damage was examined by hematoxylin and eosin (H & E) staining of formalin-fixed sections 48 hours after infection. C57BL/6 mice were inoculated with PBS (mock), *Y. pestis Y.p*91001, *Y.p*91001pPCP1^-^, *Y.p*91001 pPCP1^-^*pla*^+^ and *Y.p* 91001pPCP1^-^*pgtE*^+^ strains Representative images of inflammatory lesions are shown. **(B)** The bacteria amount in the lung tissues of the mice infected by *Y.p*91001, *Y.p*91001pPCP1-, *Y.p*91001 pPCP1-pla^+^ and *Y.p*91001 pPCP1-pgtE^+^ were examined 8 hours after infection. **P < 0.01, ***P < 0.001.

After determining the survival rate of mice, we explored whether the interaction between CD205 and Pla promotes the virulence of *Y. pestis.* We examined pathological changes in sections of infected lung histologically, using H&E staining. C57BL/6J mice were infected *via* intranasal inoculation of *Y. pestis Y.p*91001, *Y.p*91001pPCP1^-^, *Y.p*91001pPCP1^-^*pla*^+^ and *Y.p*91001pPCP1^-^*pgtE*^+^ strains. Mice inoculated with PBS were used as control. 48 hours after infection, the lung pathological changes were examined in the mice. As shown in **Figure 5A**, an influx of inflammatory cells could be detected in all lung tissue sections from infected mice. Tissue destruction and hemorrhage were more severe in 91001 wild-type- and *Y.p*91001pPCP1^−^ *pla*^+^or *pgtE*^+^-infected mice than in 91001pPCP1^−^ infected mice. However, the increasing tissue damages were most likely resulted of the increasing presences of bacteria.

The amount of bacteria in the lung tissues was determined in parallel with the data from the histological examination. *Y.p*91001pPCP1-pla^+^ is *Y.p*91001-pPCP1- restored with the plasmids pMRK1 encoding *Y. pestis* Pla, and *Y.p*91001pPCP1-pgtE^+^ is *Y.p*91001-pPCP1- restored with the plasmid pMRK3 encoding *Salmonella* pgtE. As shown in **Figure 5B**, mice infected with *Y.p*91001pPCP1-pla^+^ and *Y.p*91001pPCP1-pgtE^+^ showed exhibited a larger amount of bacteria in the lung tissues than the mice infected by 91001pPCP1−, especially the mice infected with *Y.p*91001pPCP1-pla^+^. This is consistent with the lung histological results.

Consistent with the results of the survival assay described above, we showed that the plasminogen-activating activity of Pla is essential to *Y. pestis* virulence during the injury of lung tissue.

## Discussion

In this study, we identified CD205 as a cellular receptor for PgtE, enhancing the host dissemination and infection abilities of *Y. pestis*. PgtE-expressing *Y. pestis* may utilize a similar mechanism as that demonstrated for the Pla-DEC205 interaction (32). Our data from this study indicated that PgtE and Pla shared functional similarity and were both able to bind with the C-type lectin receptor CD205. The expression of Pla and PgtE in pPCP1^−/−^ *Y. pestis* increased the mortality rate in a pneumonic plague mice model. These findings provided evidence from a functional perspective that Pla might have derived from PgtE.

The pPCP1 plasmid was acquired by an ancestral *Y. pestis* strain during the divergence from *Y. pseudotuberculosis* into modern *Y. pestis* (16). The key factor that promotes *Y. pestis* pathogenesis is plasminogen activator (Pla), which is encoded by the pPCP1 plasmid. In detail, biovar Caucasica (0.PE2) lack *pla*, *pla* in all the stains of the biovars Altaica (0.PE4), Qinghaiensis (0.PE4ab), Xilingolensis (0.PE4cd), Talassica (0.PE4), Hissarica (0.PE4), and Ulegeica (0.PE5) is the I259 phenotype. Bronze-Age (0. PRE1, 0. PRE2) (59), and Neolithic-lineage strain (6) were also express the ancestral *pla* allele (5). The most important event in the virulence optimization was the single substitution I259T occurred on the ancestral *Y. pestis* Microtus and Angola species; this increased the fibrinolytic activities of the Pla (16). In the ancestral *Y. pestis* lineages Microtus and Angola, the residue 259 in Pla is isoleucine. A single substitution I259T occurs in the ancestral lineages, in the modern lineages KIM and CO_92_ of *Y. pestis*, the position 259 is threonine (16). Cui et al. articulated that this modification of PLA may be the ‘big bang’ event during the evolution of *Y. pestis* (60, 61).

The notion, in which microbial pathogens are able to utilize the C-type lectins and the antigen presenting cells (APCs) as tools to achieve host dissemination, has started for two decades. The most established example of this process is the infection of the human immunodeficiency virus (HIV), which is mediated by the binding of HIV gp120 protein with DC-SIGN (CD209), to facilitate the infection of CD4^+^ T-cells (62–64).

In a series of our previous studies, we demonstrated that C-type lectin receptors, such as DEC-205 (CD205), Langerin (CD207), and DC-SIGN, on APCs, can bind with several Gram-negative bacteria, including *Y. pestis*, *Y. pseudotuberculosis*, and *S. enterica serovar* Typhimurium, through the core bacterial LPS, which is a key component of the outer membrane of Gram-negative bacteria, to promote bacterial dissemination (38–41, 45, 46, 48, 65–71). Our very recent report indicated that the dissemination of parasites might also follow a similar mechanism (68). These studies, particularly the 2008 report published in the Journal of Biological Chemistry (32), formed the foundation for this current study.

In the current study, two sets of *Y. pestis* with distinctively different virulence were used. One set of *Y. pestis* included *Y.p*1418, *Y.p*1419*, Y.p*1419 *pla^+^* and *Y.p*1419 *pgtE^+^*. The virulence of this set of bacteria is attenuated due to deficient pigmentation activity. The other set is the strain *Y. pestis* 91001 is, which a strain that was isolated from Microtus brandti in China, with the phenotype F1^+^/LcrV^+^/Pst^+^/Pgm^+^ (51). In short, in order to address biosafety concerns regarding different protocols, we performed the *in vivo* assay using the *Y. pestis* 91001 strains in a biosafety level III lab through collaboration with other co-authors.

Unlike the 2008 study (32), in which none of virulent strains was used, we examined whether the acquisition of PgtE would be sufficient to cause pneumonic plague following intranasal inoculation in the pneumonic plague mouse model. We infected the mice with a panel of virulent 91001 strains to examine the PgtE/mDEC-205 interaction-mediated virulence *in vivo.* Our experiments showed PgtE was in part able to rescue the competence of *Y. pestis.* This finding was consistent with the findings of a previous study, suggesting that the ancestral isolate Pestoides F, which does not carry pPCP1, was not speculated to cause primary pneumonic plague, whereas the expression of Pla in Pestoides F empowered the bacteria to cause pneumonic plague in a mouse model (16).

Our results showed that the expression of PgtE in *Y. pestis* with deleted pPCP1 was partially able to restore several functions, including plasminogen activation activity, phagocytosis by alveolar macrophages, the invasion of C-type Lectin mDEC-205-expressing cell lines, and systemic dissemination in the host. These characteristics are similar to those ascribed for Pla in previous studies from our lab and other researchers (20, 32). The ability of PgtE to restore partially the infective abilities of *Y. pestis* revealed the functional similarity between PgtE from *S. enterica* and Pla from *Y. pestis* and provided evidence to support the hypothesis that Pla in *Y. pestis* might have derived from PgtE from *S. enterica* during evolution.

CD209 and CD205 are two members of the C-type lectin receptor family, but they exhibit different characteristics. CD209 can bind with the sugar ligands from different bacteria (Gram-negative bacteria) (38–41, 45, 46, 65–70), viruses (human immunodeficiency virus) (62–64), and parasites (*Toxoplasma gondii*) (68). Thus, CD209 is a relatively unspecific receptor. In fact, the authors claim that CD209 might be responsible for almost all the so-called “non-specific binding” mediated by APCs. In contrast, CD205 can only bind with certain protein ligands, for example, the Pla of *Yersinia pestis* (32), and the pgtE of *Salmonella enterica* used in the current study. Thus, CD205 is a specific C-type lectin receptor.

As was summarized in 2006, “From an evolutionary point of view, the interaction of bacterial core LOS/LPS and the innate immune receptor, DC-SIGN, may represent a primitive interaction between microbial pathogens and the professional phagocytic host cells” (67). Moreover, in 2019, the following was stated: “We therefore propose that the loss of O-antigen represents a critical step in the evolution of *Y. pseudotuberculosis* into *Y. pestis* in terms of hijacking APCs, promoting bacterial dissemination and causing the plague” (45). Historically, CD209 has played a profound role in the evolution of pathogens, and CD205 is more specific in binding with protein ligands.

In summary, the original goal of this study was to understand of how *Y. pseudotuberculosis*, an enteric bacterial pathogen that causes only mild enteric infection, has evolved to such a deadly and distinctive pathogen, *Y. pestis*. The result provided in the study showed the PgtE from *S. enterica* can enhance the invasive abilities of *Y. pestis* through the binding of the C-type lectin receptor CD205. The CD205-PgtE interaction, similar to the CD205-Pla interaction (32), may allow the infected APCs to function as Trojan Horses, to promote dissemination within mammalian hosts and infection of *Y. pestis.* These results provide additional evidence to suggest that Pla in *Y. pestis* might have originated from PgtE in *S. enterica.* Finally, this study was initially focused on *Y. pestis*, which may however uncover for the first time one of molecular mechanisms of how *S. enterica* is able to be disseminated in the mammalian hosts.

**Table 2:** Primers and oligonucleotides used in this study.

Primer/oligonucleotide	Sequence 5′–3′
crRNA-pla top	TGGGCACATGATAATGATGAGCACTAGT
crRNA- pla bottom	TAGTGCTCATCATTATCATGTGCCCATC
pla oligo for lagging	TAATATGTTTTCGTTCATGCAGAGAGATTAAGGGTGTCTAAAAATACAGATCATATCTCTCTTTTCATCCTCCCCTAGCGG
pKD46-Cpf1-F	ACTTTGCGGCTATTCCGATGA
pKD46-Cpf1-R	TGCCGTATTGTCAGGCTCTT
pAC-crRNA-F	AGCAAGAGATTACGCGCAGA
pAC-crRNA-R	TGTAAGGGGTGACGCCAAAG
pla -WT-F	ACTATTCTGTCCGGGAGTGC
pla -WT-R	TCATGAGACTTTCCACTCAGCA
pla -deletion-F	ATTCTGTCAGACGACGAGAA
pla -deletion-R	GCGTTCCATGTCTAATTTGA

**Table 3:** Plasmids employed in this study.

Plasmid	Relevant characteristic(s)	Refs
pKD46-Cpf1-Amp	Cpf1 inserted in pKD46 using Gibson cloning, ampicillin resistance	(55, 56)
pAC-crRNA-Cm	SacB and synthetic Repeat-AcRFP1-Repeat insertedinto pACYC184 using Gibson cloning, chloramphenicol resistance	(55)
pcrRNA-pla-Cm	Protospacer of *pla* in pAC-crRNA-Cm, chloramphenicol resistance	This study
pSE380	commercially available backbone plasmid, Escherichia coli expression vector, 4476 BP, ampicillin resistance	(52)
pMRK1	the plasmid vector pSE380 carrying *pla*, with ampicillin resistance	(52)
pMRK3	the plasmid vector pSE380 carrying pgtE, with ampicillin resistance	(57)

## Data Availability Statement

The original contributions presented in the study are included in the article/supplementary material. Further inquiries can be directed to the corresponding authors.

## Ethics Statement

The animal study was reviewed and approved by Animal Care Committees of Tongji Hospital.

## Author Contributions

QL and CLY and FZ performed the experiments, analyzed the data, and wrote the manuscript. WJL, SZZ, YL, CGP, YMZ, L-YJ, KY, YXH, HHC,SZ, H-HD, OAN, JMT, and AAA assisted with the experiments. A-YL and Z-YS provided critical reagents and advice. WL, M-YY, BK, XXH, JDK, MS, APA, XFG, YPH, R-FY, XDXM, and YZW, HXC, BC provided critical reagents and advice. TC, YCS, and JPY supervised the project, designed the experiments, and modified the manuscript. All authors contributed to the article and approved the submitted version.

## Funding

This work was supported by grants from the National Natural Science Foundation of China [NSFC 81271780 and 81471915] and two local grants from Tongji Hospital, Tongji Medical College (T.C.). CP was supported by grants from the National Research Foundation of Korea [NRF-2019R1F1A1041700]. APA was supported the Ministry of Science and Higher Education of the Russian Federation [agreement number 075-15-2019-1671]. HXC was supported by Shenzhen Basic Research Project (Natural Science Foundation) Basic Research Project (No. JCYJ20190809103805589, No. JCYJ20210324112213036).

## Conflict of Interest

CP was employed by Genuv Inc.

The remaining authors declare that the research was conducted in the absence of any commercial or financial relationships that could be construed as a potential conflict of interest.

## Publisher’s Note

All claims expressed in this article are solely those of the authors and do not necessarily represent those of their affiliated organizations, or those of the publisher, the editors and the reviewers. Any product that may be evaluated in this article, or claim that may be made by its manufacturer, is not guaranteed or endorsed by the publisher.
